# Intragastrically Applicated CCl_4_-Thiopental Sodium Enhanced Lipid Peroxidation and Liver Fibrosis (Cirrhosis) in Rat: Malonedialdehyde as a Parameter of Lipid Peroxidation Correlated with Hydroxyproline as a Parameter of Collagen Synthesis (Deposition)

**DOI:** 10.5487/TR.2009.25.2.071

**Published:** 2009-06-01

**Authors:** Ki-Young Kim, Syung-Eun Cho, Byung-Soo Yu

**Affiliations:** 12 Dept. of Pathology, Medical School and Professional Graduate School of Oriental Medicine, Korea; 22 Bionanochemistry Division, Natural Science College, Wonkwang University, 344-2 IKsan City, Jeonbook, Korea

**Keywords:** Hydroxyproline, Lipid peroxidation, SOD, Collagen α1 (III) mRNA, CCl_4_-Thiopental sod

## Abstract

We investigated the pathogenesis of liver tissue damage during the lipid peroxidation and fibrogenesis with the observation of correlations between the parameters of collagen synthesis (and deposition) and lipid peroxidation in liver fibrosis (cirrhosis) rats. Rats were randomly divided into two groups, normal and CCl_4_-thiopental sod. intoxicated group. And the one group was treated intragastrically with the mixture of CCl_4_-thiopental sod. 3 times per week for 3 weeks. The liver tissue and sera were used for the measurement of hydroxyproline (HYP), malonedialdehyde (MDA) and superoxide dismutase (SOD). Biochemical parameters such as aspartate transaminase (AST), alanine transaminase (ALT), alkaline phosphatase (ALP), total-bilirubin and blood urea nitrogen (BUN) were measured. Additionally, the expression of collagen α1(III) and β-actin mRNA was observed by RT-PCR. The histological change in liver tissue was also observed by Masson’s trichrome and H&E staining. Correlation analysis was carried by Spearman’s rho method. All biochemical parameters except total-bilirubin were significantly higher in the CCl_4_-thiopental sod. treated group than that of the normal group (*p* < 0.01). In the CCl_4_-thiopental sod. treated group, Hyp as a parameter of collagen synthesis (deposition) and MDA as a metabolite of lipid peroxidation, were significantly elevated by 1.98 and 2.11 times higher than that of the normal group (*p*> 0.001) respectively. The activity of SOD in the CCl_4_-thiopental sod. treated group is decreased significantly by 44.8% (*p*> 0.001). And collagen α1(III) mRNA was more expressed in the CCl_4_-thiopental sod. treated group than that of the normal group. However, the expression of β-actin mRNA is showed similar in both of groups. A good correlation was observed between the content of hyp and MDA concentration (r = 0.70, n = 40) in the two groups. And the correlation between the levels of hyp and SOD (r = −0.71, n = 25) is also reliable. However, no correlation were observed between MDA concentration and SOD (r = −0.40, n = 25) in the two groups. Elevated levels of MDA in CCl_4_-thiopental sod. treated rats indicated enhancement of lipid peroxidation, which is accompanied by a decrease in SOD activity. Moreover, we could confirm that the parameters of collagen synthesis (and deposition) is in good correlation with the metabolite of lipid peroxidation (MDA) and the lipid peroxidation antagonizing enzyme (SOD). Hence, we propose that *➀* lipid peroxidation and collagen synthesis (and deposition) could be enhanced by intragastrically application of CCl_4_-thiopental sod. during a short terms. And ➁ the intoxication of CCl_4_-thiopental sod. could be used for monitoring of lipid peroxidation and collagen synthesis (and depositon) for test of antioxidant and antifibrotic agent.

## INTRODUCTION

The commonly used hepatotoxic agent is $CCl_4$, which transforms into the trichloromethyl free radical ($CCl_3^{\bullet}$) by cytochrome P-450 and readily reacts with $O_2$ to form trichloromethylperoxy radical ($CCl_3O_2^{\bullet}$: Parola *et al.*, 1996). Free radicals and organic radical intermediates can enhance the process of lipid peroxidation which destroys unsaturated fatty acids in cellular membranes (Schuppan, 1990), and produce toxic and reactive aldehyde metabolites such as malonedialdehyde (MDA) and HNE (4-hydroxynonenal: Poli and Parola, 1997). The other hand one of the famous reactive oxygen species (ROS) is superoxide that catalyzes the synthesis of hydrogen peroxide and oxygen by superoxide dismutase (SOD), is a living cell protect enzyme.

Except for gene expression of collagen $\alpha$1(III), as a marker of collagen synthesis, the best measurable parameter in experimental liver tissue is the hydroxyproline (hyp) content. Hyp comprises approximately 10~12% of the total amino acid in a collagen molecule. The increasing level of hyp in liver is correlated with procollagen type III peptide (Slater, 1994) or MDA/HNE (Esterbauer *et al.*, 1992; Cheeseman *et al.*, 1985) in cholestasis and alcohol induced liver disease. The major pathogenesis of tissue damage is characterized by enhanced lipid peroxidation and deranged antioxidant defensive system (Esterbauer *et al.*, 1992). The tissue fibrosis can be defined by excessive fibrogenesis (collagen synthesis) and progressive accumulation of collagens ,component of extracellular matrix proteins in various liver disease (Ruwart *et al.*, 1989; Kamimura *et al.*, 1992). But the studies of experimental liver fibrosis (cirrhosis) and manifest reports about the parameters lipid peroxidation and fibrogenesis in short term $CCl_4$-intoxication are not exist. In this report, we investigated the pathogenesis of lipid peroxidation and collagen synthesis (and deposition) with analysis correlations of parameters in normal and $CCl_4$ intoxicated rats.

## MATERIALS AND METHODS

***Reagents.*** $CCl_4$, butanol, isopropanol, methanol and chloroform were purchased from Merck (Mexico), used without purification. Thiopental sod. was received from Choongwae Pharma Co. (Korea). Chlo- ramine T, *p*-dimetylaminobenzaldehyde, SDS (sodium dodecyl sulfate), tetramethoxypropane (malonaldehyde bis(dimethyl acetal)), tetramethoxypropane, thiobarbiturate, SOD (superoxide dismutase), xanthin, XTT (2,3-bis(2-methoxy-4-nitro-5-sulfophenyl)-2H-tetrazolium-5-carboxanilide), xanthin oxidase, and other reagents were obtained from Sigma Chemical Co. (St. Louis, USA). TRIzol reagent, random hexamer, ribonuclease inhibitor, DEPC (diethyl pyrocarbonate), reverse transcriptase, 10 mM dNTP Mix. (dATP, dCTP, dGTP and dTTP), Taq. DNA Polymerase and DNA ladder were obtained from Invitrogen (TECH-LINE$^{SM}$, U.S.A.). The sense and antisense primers of collagen III and $\beta$-actin were purchased from Bioneer Co. (Korea). Biochemical parame- ters were measured with EMBIEL kit (EMBIEL LTD., Korea).

***Induction of liver fibrosis (cirrhosis).*** Female Sprague-Dawley rats (130 to 140 g) were randomly divided into 2 groups (Normal, $CCl_4$-thiopental sod. treated group) and having access to the lab chow and water under a light/dark rhythm. For the development of liver fibrosis (cirrhosis), the rats were treated intragastrically with $CCl_4$-thiopental sod. mixture (0.6 ml/rat, diluted 1 : 1 in olive oil containing thiopental sod. at a concentration of 3.6 mg/rat) three times/week for 3 weeks. The change of body weight was monitored weekly in all rats.

***Experimental sampling.*** Immediately after the sacrifice, blood was collected and centrifuged at 1,500 ×g for 10 min at 4°C and the serum was kept at -20°C until use. Liver was promptly dissected and the pieces of liver were fixed in buffered 4% formaldehyde and used for the tissue staining. The rest of liver was immediately stored at -80°C for the determination of hyp, MDA and SOD and frozen in liquid nitrogen for the observation of collagen $\alpha$1(III) mRNA and $\beta$-actin mRNA expression by RT-PCR.

***Biochemical determination.*** Aspartate transaminase (AST), alanine transaminase (ALT), alkaline phosphatase (ALP), total-bilirubin and blood urea nitrogen (BUN) were measured in sera by standard procedures with the EMBIEL-kit.

***Measurement of HYP.*** Hydroxyproline was measured by modification of Jamall's method (Jamall *et al.*, 1981). 0.2 g wet liver tissue was homogenized in 4 ml 6 N HCl and then hydrolyzed at 110°C for 12 hr. 50 $\mu$l of filtered samples in duplicate were dried, and 1.2 ml of 50% isopropanol added to dissolve. 200 $\mu$l of chloramine-T solution was added to oxidize and incubated 10 min at room temperature. 1.0 ml of Ehrlich's reagent as a chromogen was used. The mixture of all samples was incubated for 90 min at 50°C and cooled at room temperature. The absorbance of sample was read at 558 nm (Shimadzu UV-1202). The content of hyp was determined from a standard curve generated from known quantities of trans-4-hydroxyproline (0, 0.2, 0.4, 0.6, 0.8, 1.0, 1.5 and 2.0 μg/50 μl) which had been hydrolysed as described above.

***Determination of MDA.*** The concentration of MDA was determined as a lipid peroxide metabolite by the method of Okawa *et al.* (1979). 0.25 g liver tissue was homogenized in 1.75 ml of 1.15% KCl. 100 μl of 0.2% SDS were added into 200 μl of homogenates and different diluted malondialdehyde standard solution and incubated for 10 min at room temperature. Thereafter 750 μl of 20% acetic acid (pH 3.5), 750 μl 0.8% thiobarbiturate and 200 μl distilled water were added and incubated for 30 min at 95°C. After cooling on ice, 2 ml of butanol was added into the mixture and centrifuged at 1500 ×g for 5 min. The organic layer was used to measure the absorbance by spectrophotometer (Shimadzu UV-1202) at 532 nm. The results were expressed as μmol of MDA/g liver.

***Assay of SOD activity.*** 0.1 g liver tissue was homogenized in 2.0 ml 50 mM Tris buffer (pH 7.6) containing 0.1 mM EDTA and centrifuged at 3500 ×g for 10 min at 4°C. The supernatant was directly extracted with a chloroform and ethanol mixture (37.5 : 62.5 v/v), and the suspension was centrifuged at 200 ×g for 10 min at 4°C. The supernatant was used for determination of SOD by the modified method of Okado-Matsumoto *et al.* (2001). 50 μl of diluted sample solution and diluted SOD standard solutions were added into a mixture with 1.25 ml of 50 mM sodium phosphate buffer (pH 8.0), 50 μl each of 3 mM EDTA, 3 mM xanthine and 0.75 mM XTT solution. The reaction was initiated by the addition of 50 μl of 200 mU/ml xanthine oxidase solution. After 10, 20, 30 min incubation at 30°C in a water bath, the absorbance change was measured at 470 nm by spectrophotometer. The results were plotted as IU SOD/0.1 g liver.

***Collagen α1(III) and β-actin mRNA expression by RT-PCR.*** Total RNA was extracted from liver tissue by using the TRIzol reagent. About 2 μg of total RNA were reverse-transcribed into complementary DNA (cDNA). A set of primers (Kauschke *et al.*, 1999) for the collagen α1(III) and β-actin gene which give an amplified fragment of 295 bp and 250 bp, resp. were used. The reaction mixture for the PCR consisted of 5 μl of ten-fold PCR buffer, 1.5 μl of 50 mM $MgCl_2$, 1.0 μl of 10 mM dNTP, 35 μl of DEPC $H_2O$, 0.5 μl of Taq. polymerase, 1 μl each of a sense (CGC GGG TAC CCT ATG GCA ATC CTG ATC TTC CTG A) and antisense primers (CGC GAA GCT TCA TGG CCT GTG TTT GA) of collagen III and β-actin, 2 μl of synthesized cDNA solution, providing a total volume of 50 μl. After incubation at 94°C for 5 min, the reaction mixture were denatured at 94°C, primers were annealed at 58°C, and DNA synthesis took place at 72°C (each step 45 sec, 30 cycles, Eppendorf, Master cycler gradient, USA). At the end an additional extension time of 5 min at 92°C was performed. Subsequently, the reaction was paused at 4°C. Reaction mixture and 50 bp DNA ladder as DNA size markers were resolved by electrophoresis on 2.0% agarose gel with TBE buffer for ethidium bromide staining. Band intensities of amplified β-actin and collagen α1(III) cDNAs were compared after visualization on a UV transilluminator.

***Histological procedures.*** For histological examination, liver specimens were fixed in 4% buffered formaldehyde, and embedded in paraffin and stained with hematoxylin-eosin and Masson's trichrome. The histological changes such as focal necrosis, lipid droplets, inflammation cell and collagen fibers were observed in portal triads and stroma.

***Statistical analysis.*** The significance of all results was tested with Student's *t*-test and expressed as *p*-value. Data show the mean ± standard deviations. Correlation-analyses of each parameter was also evaluated by Spearman's rho method.

## RESULTS

***Change of liver weight and the ratio of liver weight/body weight.*** Table 1 shows decreased body weight, significantly higher liver weight and the ratio of

**Table 1.** The weight of body, liver and the ratio of liver/body weight in normal and liver fibrosis-induced rats

| Group | Body weight (g) | | Liver weight (g) | Ratio of LW/BW (%) | n |
|---|---|---|---|---|---|
| | 0 week | 3 week | | | |
| Normal | 134.9 ± 6.4 | 193.5 ± 9.3 | 6.36 ± 0.46 | 3.29 ± 0.30 | 21 |
| AC | 136.2 ± 7.4 | 169.1 ± 12.3* | 12.98 ± 1.19** | 7.68 ± 0.60** | 28 |

Data are expressed as mean ± SD, * Significantly different from normal group ($p < 0.005$), ** Highly significantly different from normal group ($p < 0.001$), AC: $CCl_4$-thiopental sod. treated group. LW/BW: liver weight/body weight, n: number of animals.

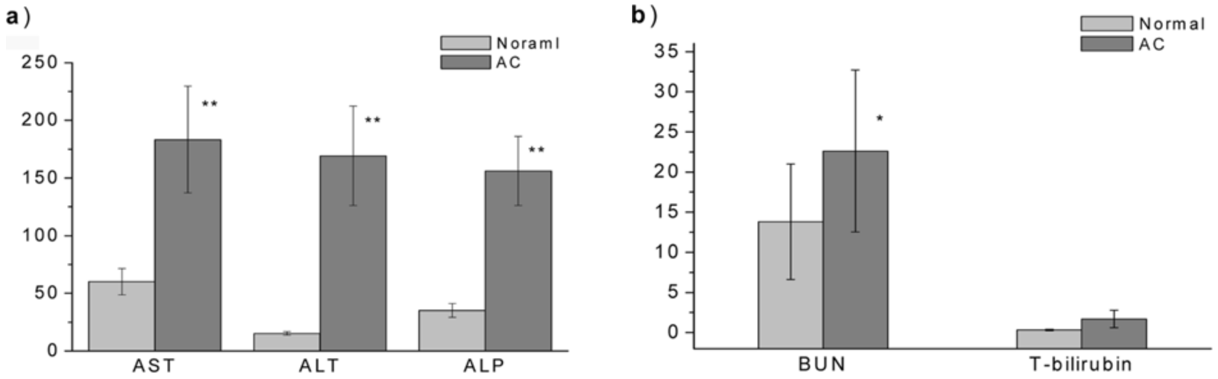

**Fig. 1.** The biochemical parameters in sera of normal and fibrotic (cirrhotic) rats. a) AST, ALT and ALP; b) BUN and total-bilirubin. Data are expressed as mean ± SD, *: significantly different from normal group ($p < 0.05$); **: highly significantly different from normal group ($p < 0.001$); AC: CCl4-thiopental sod. treated group.

**Table 2.** Content of HYP and MDA and activity of SOD in liver tissue of normal and fibrotic (cirrhotic) rats

| Group | Hydroxyproline (μg/g liver) | n | Malonedialdehyde (nmol/g liver) | n | Superoxide dismutase (IU/g liver) | n |
|---|---|---|---|---|---|---|
| Normal | 359.0 ± 76.5 | 21 | 169.5 ± 26.6 | 21 | 142.7 ± 32.8 | 13 |
| AC | 1145.7 ± 364.1* | 28 | 307.1 ± 90.3* | 28 | 65.6 ± 17.7* | 19 |

Significantly different from normal group ($p < 0.005$), AC: $CCl_4$-thiopental sod. treated group, n: number of rats.

liver weight/body in $CCl_4$-thiopental sod. treated rats than that of normal rats ($p < 0.005$).

***Clinical biochemical parameters.*** Standard liver function tests were analyzed for monitoring of necrosis and liver damage. Significantly increased value of AST and ALT were observed in $CCl_4$-thiopental sod. treated rats ($p < 0.001$, Fig. 1a). In addition, the levels of BUN and ALP were significantly greater in $CCl_4$-thiopental sod. treated rats than that of normal rats ($p < 0.05$~0.001, Fig. 1a) by 4.44 and 4.88 times. Total-bilirubin in $CCl_4$-thiopental sod. treated rats was higher than that of normal group, but showed no significance (Fig. 1b).

***Collagen deposition in liver tissue.*** The content of hyp in liver tissue is a parameter of collagen deposition and collagen imbalance. The value of hyp is significantly elevated (1.98 times) in the $CCl_4$-thiopental sod. treated group compared to normal group ($p < 0.001$, Table 2).

***Lipid peroxidation-derived aldehyde.*** Activated lipid peroxidation in liver results in liver cell damage and enhanced fibrogenesis. The concentration of MDA as a lipid peroxidation metabolite was 2.11 times higher in $CCl_4$-thiopental sod. treated group than that of normal group ($p < 0.001$, Table 2).

***SOD activity.*** One of the major protective enzyme in living cell is SOD. The concentration of SOD is significantly decreased (48.2%) in the $CCl_4$-thiopental sod. treated group compared to the normal group ($p < 0.001$, Table 2).

***Collagen $\alpha$1(III) and $\beta$-actin mRNA expression.*** The expression of β-actin mRNA which was used as a housekeeping gene was almost similar in both group (Fig. 5). However collagen α1(III) mRNA mRNA was more expressed in the $CCl_4$-thiopental sod. treated group than that of normal group ($p < 0.001$, Fig. 2).

***Relationships among hyp, MDA and SOD.*** The content of hyp were correlated with MDA concentration (r = 0.70, n = 40) or SOD (r = -0.71, n = 25), while very weak negatively correlation were observed between MDA and SOD (r = -0.40, n = 25) as shown in Fig. 3.

***Histological change.*** Focal necrosis, excessive lipid droplets in periportal triads and stroma, thick col-

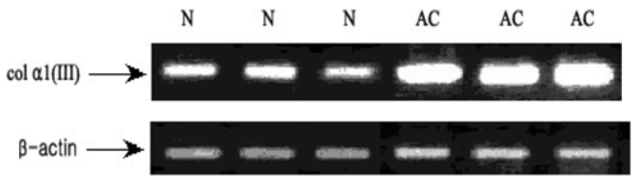

**Fig. 2.** Gene expression of collagen α1(III) and β-actin in normal and fibrotic (cirrhotic) livers by RT-PCR N: normal rat, AC: $CCl_4$-thiopental sod. treated group.

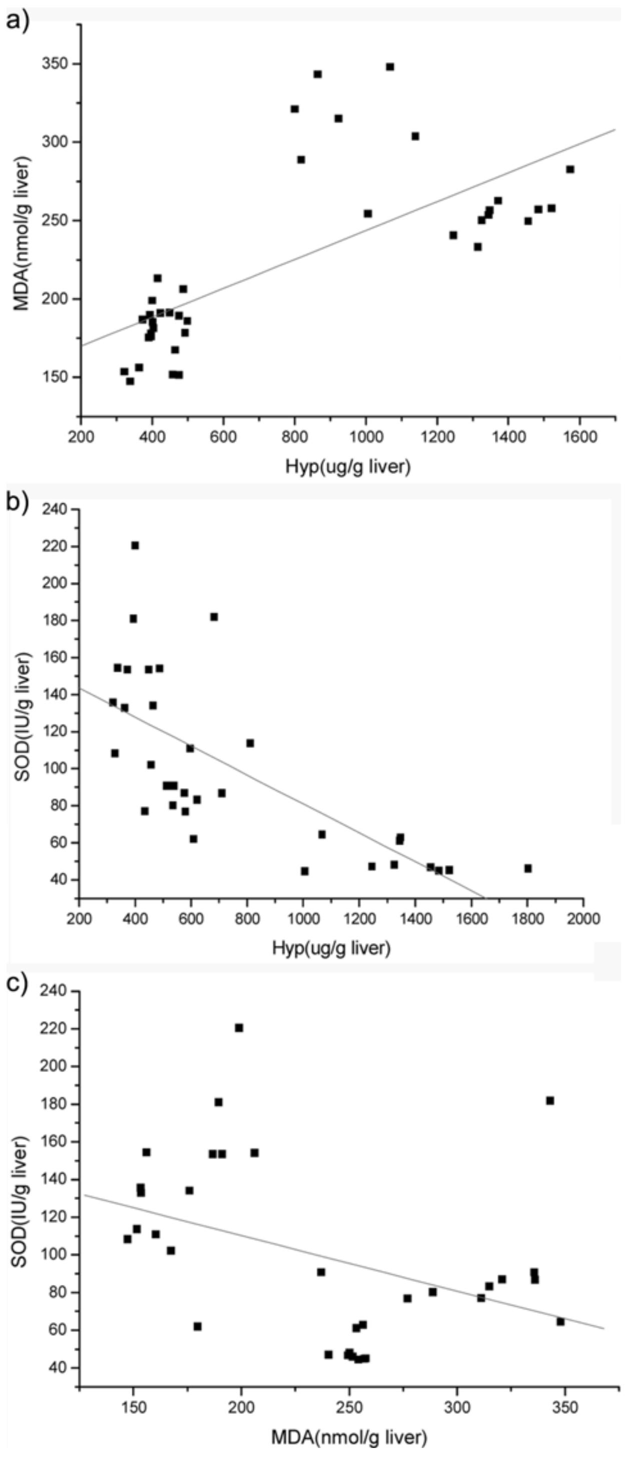

**Fig. 3.** Diagram of correlation between the HYP, MDA and SOD in normal and liver fibrosis (cirrhosis) rats. a: Good correlation between HYP and MDA ($r = 0.70$, $n = 40$), b: good correlation between HYP and SOD ($r = -0.71$, $n = 25$); c: weak correlation between MDA and SOD ($r = -0.40$, $n = 25$).

lagen fiber in portal triads, lysis of cytoplasma and crashed down nuclei were observed in $CCl_4$-thiopental sod. treated group by staining with Masson's trichrome. Moreover, collagen fibers bridged one portal and neighbor portal area and appearance of inflammatory cells were observed in $CCl_4$-thiopental sod. treated group (Fig. 4).

## DISCUSSION

Oxidative stress has been shown to enhance lipid peroxidation that has been indicated relationship between chronic liver damage and fibrosis (Cheeseman *et al.*, 1985; MacDonald *et al.*, 2001; Vengerovskii *et al.*, 1996).

In this study, the pathogenesis of liver tissue damage was investigated by the observation of correlation between the parameters of collagen synthesis (and deposition) and lipid peroxidation in liver fibrosis (cirrhosis) induced rats treated a short term with $CCl_4$-thiopental sod. Our study showed that the value of MDA as a product of lipid peroxidation in liver tissue and the level of AST and ALT in sera of $CCl_4$-thiopental sod. treated group as a biochemical parameter of necrosis were significantly increased ($p < 0.005$, Fig. 1a). Surveying the studies of the past, a long term $CCl_4$-intoxication could be induced lipid peroxidation and liver damage, and thereby appeared increased levels of MDA, AST and ALT (Gasso *et al.*, 1996; Hernandez-Munoz *et al.*, 1997), but the change of SOD activity is highly disputed (Hernandez-Munoz *et al.*, 2001; Cabre *et al.*, 2000). Additionally, increased product of lipid peroxidation (MDA, HNE) were reported not only in the plasma of patient with decompensated liver cirrhosis and in hepatitis C liver tissue (Chen *et al.*, 1997; Paradis *et al.*, 1997), as well as in the liver tissue of animals with ethanol, dimethylnitrosamine, or iron overload induced liver damage (Bacon and Britton, 1990; Niemelae *et al.*, 1995; Vendemiale *et al.*, 2001). Our results shows enhanced lipid peroxidation and necrosis were developed by a short term $CCl_4$- thiopental sod. intoxication.

Nadkarni and D'Souza reported that only the concentration of SOD, which had been shown to antagonize lipid peroxidation, in $CCl_4$-phenobarbitone treated rats. However no increasing in hepatic lipid peroxidation metabolite (MDA) was observed. These results suggests that in cirrhotic livers cell damage are resulted by the direct attack of oxygen free-radicals (Nadkarni and D'Souze, 1998). Henandez-Mundez *et al.* reported that activity of SOD after 4 and 8 weeks $CCl_4$-treatment was similar in the normal and $CCl_4$-intoxificated group (Hernandez-Munoz *et al.*, 1997).

In the current study, the concentration of SOD in the $CCl_4$-thiopental sod. treated group was decreased by

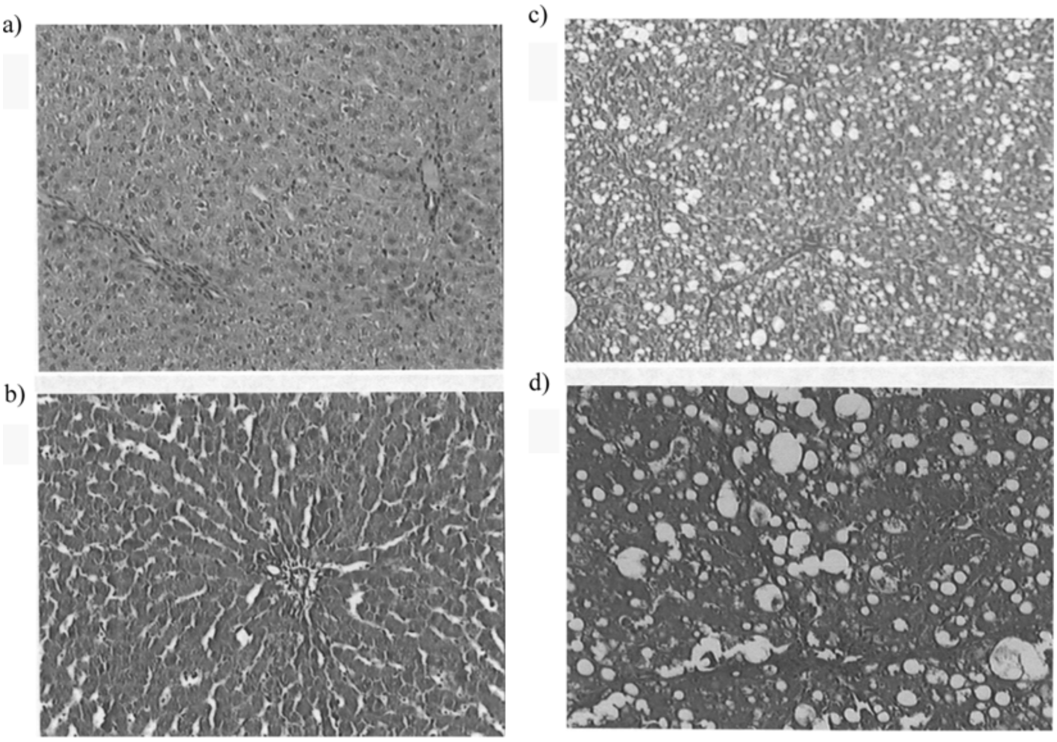

**Fig. 4.** Histological change of liver in normal and fibrotic (cirrhotic) liver of rats. a, b: Central vein and portal triad in the normal liver of rats (a: H&E staining, × 120; b: Masson's trichrome staining, × 120); c, d: focal necrosis, lipid droplets, portal-portal and portal-central septum and collagen fibers in portal area and stroma of $CCl_4$-thiopental sod. treated rat (c: H&E staining, × 80; Masson's trichrome staining, × 120).

44.8%, showing a similar trend as in Nadkarni's report (Nadkarni and D'Souza, 1998), on the other hand the lipid peroxidation product (MDA) was increased 2.11 times (Table 2). The differential results can be bring down to various influence factors. The administration method of $CCl_4$ could be the one of the factors. Nadkarni and D'Souza used $CCl_4$–gassing plus phenobarbitone in drinking water. In our study, we used a mixture of $CCl_4$–thiopental sod. intragastrically.

From this argument, our data could be indicated that liver damage may be induced by direct attack of free radical and thereby lipid peroxidation stimulated and the balance of antioxidant defensive system might be broken. Also we observed that the expression of collagen $\alpha$1(III) mRNA/$\beta$-actin mRNA was increased in $CCl_4$- thiopental sod. treated group than that of normal group (Fig. 2). Similar result was reported that increased in the expression of collagen $\alpha$1(III) mRNA is an indicator of collagen synthesis by quantitative RT-PCR in dimethylnitrosamine induced liver fibrosis (Shiba *et al.*, 1998) with $CCl_4$- induced liver fibrosis (Kauschke *et al.*, 1999). And increased concentration of MDA and expression of collagen $\alpha$1(III) mRNA were related with activation of HSC (Lee *et al.*, 1995; Maher *et al.*, 1994).

Our data demonstrated that lipid peroxidation could play a key role in fibrogenesis of liver disease as reported (MacDonald *et al.*, 2001; Ljubuncic *et al.*, 2000; Rhoden *et al.*, 2000). It might act as a link between tissue injury and liver fibrosis by modulating collagen gene expression (MacDonald *et al.*, 2001; Ljubuncic *et al.*, 2000). A lot of research about good correlation between the parameters of lipid peroxidation, fibrogenesis, and collagen deposition presented e. f. MDA correlated with the score of fibrosis in liver tissue of patient with hepatitis C (Paradis *et al.*, 1997). In gluthation peroxidase in sera of $CCl_4$-treated rats was also reported ($r = -0.47$, $p < 0.001$) (Cabre *et al.*, 2000). And hyp in liver tissue as an indicator of collagen synthesis (deposition) correlated with 4-HNE ($r = 0.81$, $p < 0.001$) or MDA ($r = 0.71$, $p < 0.001$) in sera of cholestasis-induced rats has been reported (Esterbauer *et al.*, 1992; Rhoden *et al.*, 2000).

Our data showed good correlation between hyp and MDA ($r = 0.70$, $n = 40$) in liver tissue of normal and $CCl_4$-thiopental sod. treated. The SOD activity correlated negatively with hyp ($r = -0.71$, $n = 25$) and with MDA ($r = -0.40$, $n = 25$). As a result, the treatment with $CCl_4$-thiopental sod. enhanced free radical generation, stimulated excessive lipid peroxidation and enhanced collagen synthesis and deposition.

It was reported in minipig with ethanol-induced liver disease that products of ethanol metabolism such as acetaldehyde and the metabolite of lipid peroxidation (MDA) appeared to precede necrosis and fibrosis (Niemelae *et al.*, 1995). Similar results were observed in our study. Thus the values of ALT and AST as an indicator of necrosis were higher in $CCl_4$-thiopental sod. treated group than that of the normal group ($p < 0.05$~0.001, Fig. 1). The necrosis and fibrosis of liver tissue were observed by Masson's trichrome staining in $CCl_4$-thiopental sod. treated group (Fig. 4). In addition, a significantly higher liver weight and enhanced ratio of liver/body weight as a sign of hepatomegaly were induced by $CCl_4$-thiopental sod. intoxication ($p < 0.001$, Table 1).

In conclusion, elevated levels of MDA in $CCl_4$-thiopental sod. treated rats indicated enhancement of lipid peroxidation, which is accompanied by a decrease in SOD activity. Moreover, we could confirm that the parameters of collagen synthesis (and deposition) is in good correlation with the metabolite of lipid peroxidation (MDA) and the lipid peroxidation antagonizing enzyme (SOD). Hence, we propose that lipid peroxidation and collagen synthesis (and deposition) could be enhanced by intragastrically application of $CCl_4$-thiopental sod. during a short terms. And the intoxication of $CCl_4$-thiopental sod. could be used for monitoring of lipid peroxidation and collagen synthesis (and depositon) for test of antioxidant and antifibrotic agent.

## ACKNOWLEDGEMENTS

This paper was supported by 2007 Wonkwang University.
